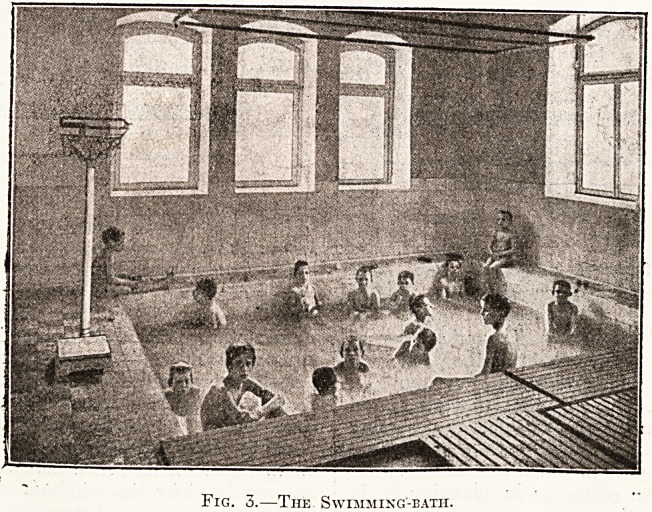# A German School of Recovery

**Published:** 1914-02-14

**Authors:** 


### A GERMAN SCHOOL OF RECOVERY.

### The Example of the Mannheim Municipality.

Some months ago (The Hospital, May 10, 1913) we gave an account of the Ogilvie School of Recovery at Clacton-on-Sea. A comparison of this home with a newly established German home will therefore not be without interest.

The Victor Lenel Institute in the Neckarthal, near Mannheim, is the gift of Councillor Victor Lenel to his native city, Mannheim, for the purpose of providing a school of recovery for children attending the elementary and secondary schools in the city. It is built at an elevation of 600 feet above the sea-level, in a picturesque little valley, and was designed by architect Koechler. Ample play-room is provided on a site which totals 700 square metres; water is derived from a local spring which gives more than sufficient for the somewhat extravagant demands of the home. The orientation of the building is, as may be seen from the plan, such that the rooms get the maximum of sunlight. Simplicity, easy access to all parts, roominess, and efficiency have been the guiding lines in designing the home. The ground-floor plan will give some indication of the manner in which certain structural difficulties have been overcome in planning. It sufficiently explains the arrangement of rooms on this floor; we may add that the reading-room is also used as a class-room, and that excellent provision is made on all floors to cope with an outbreak of fire. Each floor contains a double lavatory and w.c. with two small and one large closet. The staircase is broad and easy and well guarded. On the first floor are the matron's quarters; these are well arranged and consist of a sitting-room (17 by 11 feet), a bedroom (15 by 12 feet), and a bath and lavatory room (10 by 7 feet). On the other side is a ward with lavatory and a room for the ward nurse. On the south-east side are two large dormitories, each 38 by 22 feet, with two small sleeping-rooms attached for the dormitory nurses. Each dormitory has a wide balcony, and the estimate per bed is 3.35 square metres floor surface and 15 cubic metres air space. Two sizes of bedstead are provided; these are of white enamelled iron, with wire mattresses on which are laid coir (for children suffering from incontinence) or horsehair mattresses. The bed coverings are ample, the outer covering being a white and coloured counterpane which gives the bed an attractive appearance. Alongside each bed is a small white enamelled chair for the child's underclothing; the other clothing is removed outside the dormitory and hung on numbered pegs. Each dormitory is provided with excellent lavatory accommodation; each child has his or her own towel, soap, brush and comb, toothbrush, sponge, and washing glove. A separate bathroom is provided on this floor for medicinal baths. The dormitories are ventilated by the windows and are heated by low-pressure steam radiators.

Fig. 1. Ground-floor Plan.
1. Dining-room. 2. Office. 3. Visitors' room. 4. Playroom. 5. Reading-room. 6. Sewing-room. 7. Observation-room (for sick children). 8. Lavatories. 9. Pantry. 10. Kitchen and scullery. 11. Serving-room and Servants' Dining-room.

On the second floor are two other dormitories, each accommodating twenty children; these rooms have lavatories and nurses' rooms attached. A bathroom is provided on this floor for the use of nurses. On this floor is also the cook's bedroom, with a three-bedded room for nurses and a four-bedded dormitory for servants, for whom separate lavatory accommodation is provided.

The flooring throughout is of inlaid linoleum, except in the lavatories, where terrazo is employed. The various dormitories and living rooms are made bright and attractive by the use of a light-coloured wall paint, relieved by white enamel, so that the impression made on the mind of the visitor is that

Fig. 2.—The Victor Lenel Institute. Exterior View.

cleanliness is one of the main tenets of the administration. One so often sees, in schools and children's homes, that a dark-looking wall paint is used, the excuse being that "it doesn't show the dirt so much"; here, at least, the principle that dirt should not be hidden but removed has been acknowledged with eminently satisfactory results.

The basement floor is unusually interesting. The building is placed on a slight slope, so that part of this floor is above ground and part of it below. The main entrance to the basement is in that portion of it which is above ground. Next to the entrance hall is a cloak-room, and alongside of it is a special boot-room, the children being required to take off their boots when they enter from play and put on special "house shoes." From the hall one enters a beautifully light playroom, 37 by 22 feet, which also serves as the boys' gymnasium, and which gives access to a fine terrace overlooking the valley. In the western extremity of this floor is the swimming-bath, 9½ square metres in area and 50 centimetres deep. Alongside it is the room for the house master. The subterranean portion of the basement floor contains the cellarage, a milk-room, a laundry-room, and an extra room. The boiler-house is placed below the gymnasium.

A 500 square metre playground has been provided, and a kitchen garden and orchard have been added. The home was opened on May 15, and three days later received its first batch of children. Now it accommodates ninety children, boys and girls, between the ages of six and fourteen. The matron, Frau Zentmayer, is a trained head teacher, and there are six assistant teachers, four of whom are volunteers who give part of their time to the work, a head and an assistant nurse. The personnel consists of a cook, four housemaids, and a "housemaster," who, however, does not concern himself with pedagogic work but supervises the boiler-house and garden. The home is under the control of the Mannheim school medical officer, but its medical supervision is entrusted to a local doctor who visits the children from time to time and reports on each new comer. The house rules are interesting. The children are never awakened in the morning. The ward teacher comes into the dormitory at seven o'clock in summer and eight o'clock in winter, when those children who are already awake may get up; by and by the others also awake and get up. After morning ablutions all the children go into the open air in fine weather, or into the large playroom if the weather is bad, and indulge in breathing exercises. After breakfast the children make their beds and tidy the dormitories. Then they are grouped into various companies; some clean the boots, some

Fig. 3.—The Swimming-bath.

work in the garden, some go swimming, and others to lessons in the reading-room or in the open. Five boys and five girls, volunteers from among the older children, have to see that the day-rooms are clean and that flowers and foliage are fresh in the dining-hall. A light meal is served at eleven o'clock, and at one dinner is served. After this meal a period of rest on the balcony or in the playground is enforced. After this comes bread and butter with milk or malted coffee; then a further term of activity until supper is served at half-past six or seven. At nine o'clock all lights are out and everyone is abed. The total expenses of the home per year average £1,200. Each child pays one shilling a day, or if his parents or friends cannot afford to pay for him the municipality does so.

The Victor Lenel Institute is one of the most interesting experiments on the Continent and well repays a visit by those who wish to see for themselves what such a home, under good management, can produce. For the illustrations we are indebted to the excellent report of the School Medical Officer for Mannheim, Dr. P. Stephani, which contains a full description of the home and a copy of its rules.

## Figures and Tables

**Fig. 1. f1:**
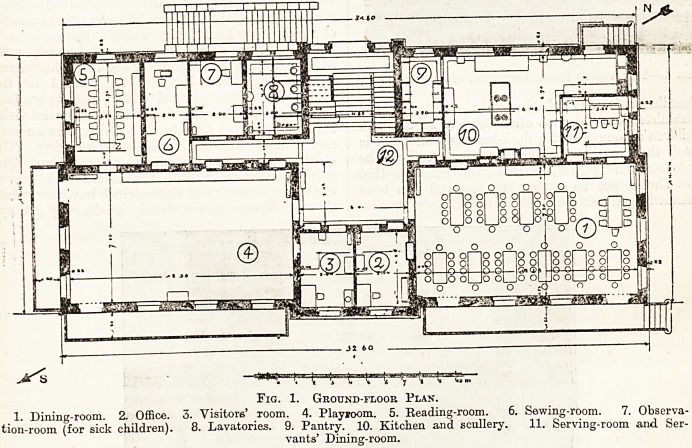


**Fig. 2. f2:**
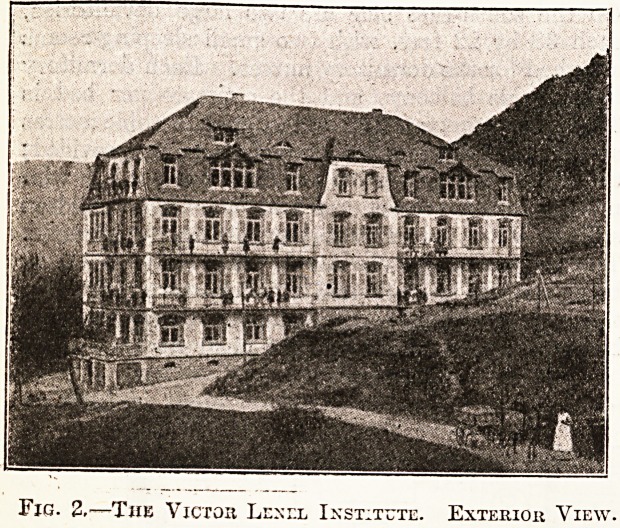


**Fig. 3. f3:**